# Cumulative incidence of chronic health conditions recorded in hospital admissions from birth to age 16 in England: a cohort study using linked hospital and education administrative data

**DOI:** 10.23889/ijpds.v9i5.2487

**Published:** 2024-09-18

**Authors:** Helena Benes Matos da Silva, Juliana Freitas de Mello e Silva, Rita de Cássia Ribeiro Silva, Irina Chis Ster, Poliana Rebouças, Emanuelle Goes, Maria Yury Ichihara, Andrêa Ferreira, Julia M. Pescarini, Rosemeire Leovigildo Fiaccone, Enny S. Paixão, Maurício L. Barreto, Bethania de Araujo Almeida

**Affiliations:** 1 School of Nutrition, Federal University of Bahia; 2 Center for Data and Knowledge Integration for Health; 3 Infection and Immunity Research Institute, St George’s University of London; 4 Epidemiology and Population Health, London School of Hygiene and Tropical Medicine; 5 Department of Statistics, Federal University of Bahia; 6 Núcleo de Pesquisa

Growth is an important marker of child health and development. It is related to aspects such as social-economic conditions, being affected by social inequalities. Race is a social construct that functions as an essential tool of racism. It creates social hierarchy, resulting in segregation, different quality and access to health care, and unequal distributions of social determinants on health. Understanding the effects of ethno-racial inequalities on growth trajectories is essential to improve children development and well-being. In the study we investigated child growth according to maternal ethno-racial group using a nationwide Brazilian database. Data were obtained via linkage of the CIDACS Birth Cohort and the Brazilian Food the Nutrition Surveillance System. It included 4,090,271 children under 5 years old, born at term, followed-up between 2008 and 2017. We used mixed-effects model to estimate both original and standardized forms of length/height and weight trajectories. Results pointed out to growth differences among the racial groups, concerning both markers. Children born to Indigenous mothers presented the most critical situation, followed by those born to Parda, Asian-descent, and Black mothers. The strengthening of policies aimed at protecting Indigenous children should be urgently undertaken to address systematic ethno-racial health inequalities. Additionally, these findings may help policymakers to achieve the United Nation’s 2030 Sustainable Development Goals (eradication of hunger and all forms of malnutrition).

